# The PDZ motif peptide of ZO-1 attenuates *Pseudomonas aeruginosa* LPS-induced airway inflammation

**DOI:** 10.1038/s41598-020-76883-9

**Published:** 2020-11-12

**Authors:** Tae Jin Lee, Yung Hyun Choi, Kyoung Seob Song

**Affiliations:** 1 Department of Anatomy, College of Medicine, Yeungnam University, Nam-gu, Daegu, Korea; 2 Department of Biochemistry, College of Korean Medicine, Dong-Eui University, Busan, Korea; 3 Department of Cell Biology, Kosin University College of Medicine, 34 Amnam-dong, Seo-gu, Busan, 49267 Korea

**Keywords:** Biochemistry, Physiology, Molecular medicine

## Abstract

*Pseudomonas aeruginosa* is known to play a role in many human diseases. Therefore, examining the negative control mechanisms of tight junction protein ZO-1 on the exotoxin LPS of *P. aeruginosa*-induced diseases could be critical in the development of novel therapeutics. We found that ZO-1 expression dramatically decreased in inflammatory human lung tissues. Interestingly, PDZ1 deletion of the PDZ domain in the ZO-1 protein dramatically decreased LPS-induced F-actin formation and increased the expression of genes for pro-inflammatory cytokines, but not PDZ2 and PDZ3 of the ZO-1 protein. We also found that the consensus PDZ peptide (based on PDZ1) of ZO-1 down-regulates the expression of *pro-inflammatory* cytokine genes and F-actin formation; in contrast, the GG24,25AA mutant PDZ peptide cannot control these genes. LPS activates IL-8 secretion extracellularly in a time-dependent manner, while the secretion is inhibited by PDZ peptide. Whereas increased IL-8 secretion by LPS activates the CXCR2 receptor, overexpressed RGS12 negatively regulates LPS-induced CXCR2/IL-8 signaling. The PDZ peptide also decreases LPS-induced inflammatory cell populations, pro-inflammatory cytokine gene expression, and TEER in bronchoalveolar lavage fluid and cultured alveolar macrophages. Collectively, we suggest that the PDZ peptide may be a potential therapeutic for bacteria-induced respiratory diseases.

## Introduction

*Pseudomonas aeruginosa* is an ordinary gram-negative, encapsulated, rod-shaped bacterium that can induce severe diseases in humans. In particular, respiratory infection is representative of a major clinical problem globally and has major consequences for patients^1^. Respiratory infections are more lethal for patients with inflammatory conditions, such as asthma or COPD, therefore, studies examining the negative control mechanisms of *P. aeruginosa*-induced diseases are critical to the development of novel therapeutic medications. Moreover, pseudomonal infections are becoming progressively resistant to some antibiotics and may gain resistance during therapy. *P. aeruginosa* has become a critical problem causing 51,000 healthcare infections in the USA per year^2^. Because of limitations in the current therapy (β-lactams, aminoglycosides, carbapenems, colistin, or the combination of multiple antibiotic classes)^2^, alternative drugs and new therapeutic options need to be developed to decrease antibiotic resistance.

The damage to the protective role of mucosal barriers that network with the inflamed environment is necessary for the development of autoimmunity^3^. Tight junction proteins consist of zonula occludens (ZO) 1, 2, 3, occludin, and claudins, which control the paracellular permeability of apical and basolateral membranes, and function as a wall to prevent bacterial attack^4^. Damage to the tight junction complex could induce many diseases. In asthma and cystic fibrosis patients, one of the negative effects of chronic airway inflammation is hypersecretion and intercellular tight junction protein disruption in epithelial cells^5–7^. Even in structural duplication, the specific functions of ZO-family proteins are not actually overlapped and they have embryonic lethality in mice lacking either ZO-1 or ZO-2, but not ZO-3 ^8–10^. The levels of paracellular barrier and actin re-organization are decreased in ZO-1 knockdown cells^10^.

IL-8 (CXCL8) is a typical member of the subfamily of N-terminal Glu-Leu-Arg (ELR) motif-containing CXC chemokines that activates inflammation by accumulating neutrophils^11^. The signal mechanisms are associated with the interaction of CXCL8 with CXCR1 and CXCR2 receptors, which are G-protein coupled receptors (GPCRs). Following CXCR1/2 activation, GqPCR-induced IP3 and Ca^2+^ signaling has been demonstrated in humans^12^. Of the mechanisms for GPCR desensitization, the regulators of G-protein signaling (RGS) family are negative regulators of specific GPCR signals^13,14^. RGS proteins can accelerate the GTPase activity of the GTP hydrolysis activity of G-protein α subunits, suggesting that RGS proteins change GTP-bound Gα to an inactive GDP-bound form to down-regulate GPCR signaling^15^. However, the mechanisms by which RGS proteins can desensitize the CXCR2-induced pathway activated by components of *P. aeruginosa* remain to be examined.

Here, the aim of this study was to identify whether a component of ZO-1 tight junction protein or itself has an anti-inflammatory effect against *P. aeruginosa* in the airway. Whereas overexpressed ZO-1 did not regulate LPS-induced inflammation, the PDZ1 domain of ZO-1 dramatically decreased LPS-induced inflammation in vitro and in vivo. Interestingly, the PDZ peptide suppressed LPS-activated inflammatory cell populations and pro-inflammatory cytokine gene expression in bronchoalveolar lavage fluid (BAL) fluid and cultured alveolar macrophages (AM) cells. Our data indicate that the PDZ domain in ZO-1 is critical for the regulation of the LPS-induced inflammatory microenvironment.

## Results

### ZO-1 expression is decreased in human lung diseases related to inflammation

**Figure 1:**
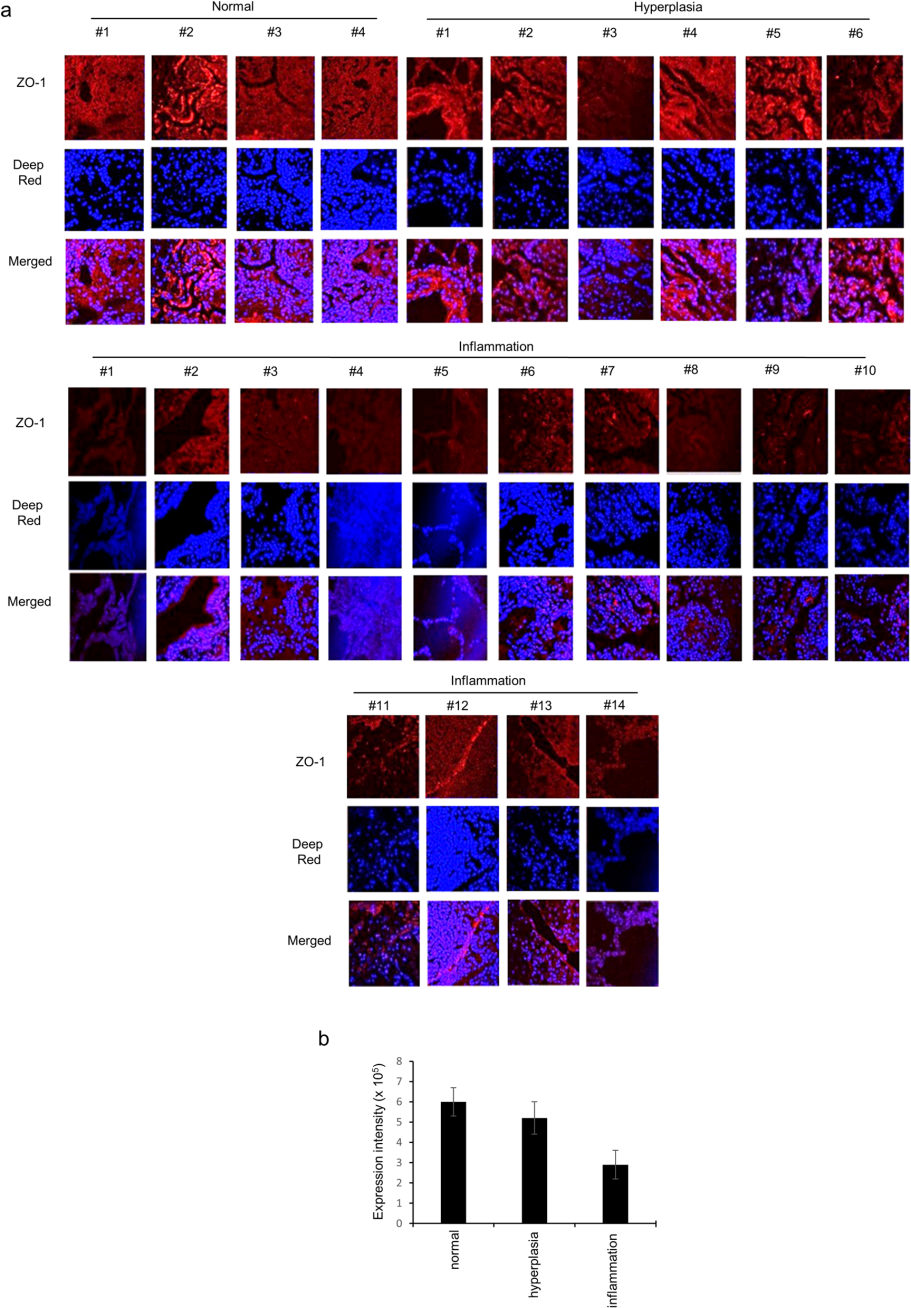
ZO-1 expression in human pulmonary diseases related to inflammation. **(a)** Three different tissues from normal, hyperplasia, and inflammation groups were used. Lung sections were processed with anti-ZO-1 antibody, stained with Deep-Red, and images were obtained for each core. Five separate images of each section were given a score (1– 7) by three independent scientists. **(b)** The expression density was used to assess stained differences between cells. *p < 0.05 compared with normal. All data shown are representative of three independent experiments.

To discover whether ZO-1 expression is clinically associated with inflammation in human lung tissue, a human pathological lung tissue array was assessed for ZO-1 expression (Fig. 1). Whereas ZO-1 expression was detectable in normal tissues, it was dramatically decreased in the inflammation group and was slightly decreased in the hyperplasia group. Representative images are shown with disease quantification scores (Fig. 1b). The lung pathologies suggest that a significant decrease in ZO-1 expression is closely related to both disease processes.

### PDZ domain of the ZO-1 protein is critical for LPS-induced F-actin formation

**Figure 2:**
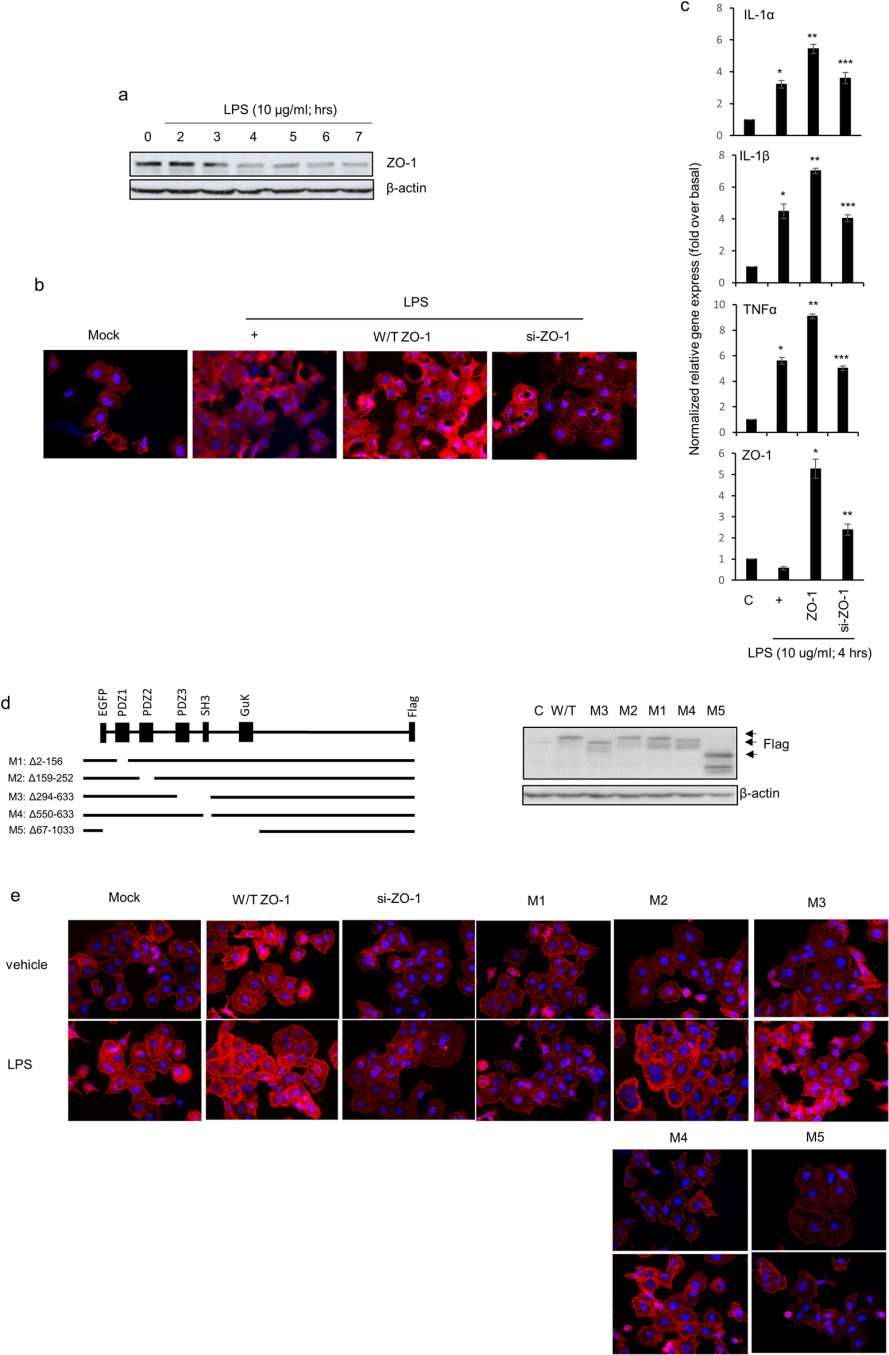
The first PDZ motif of ZO-1 protein attenuates LPS-induced F-actin formation. **(a)** BEAS-2b were treated with LPS (10 µg/ml) in a time-dependent manner. β-actin was used as a loading control. **(b)** NCI-H292 cells were transfected with either a construct driving the expression of wild-type ZO-1 or ZO-1-specific siRNA. Cells were then incubated with LPS for 10 h. The cells were stained with ActinGreen 488 ReadyProbe reagent. **(c)** Cells were transfected and were then incubated with LPS for four hours before the generation of total cell lysates, and pro-inflammatory cytokine transcripts were assessed by qRT-PCR. *p < 0.05 compared to the control, **p < 0.05 compared to LPS only, and ***p < 0.05 compared to ZO-1-transfected cells. **(d) **Constructs were designed according to the amino acids deleted (e.q. M1: 2–156). Black lines represent the magnitude of the sequences encoded by each construct. The expression level of each construct was checked by Western blotting (anti-Flag; right panel). **(e)** The cells were transfected with either a ZO-1 overexpression construct or deletion constructs and incubated with LPS for 10 h. F-actin was stained with a specific reagent.

To investigate whether LPS could regulate ZO-1 expression at an early event, LPS was treated in a time-dependent manner in BEAS-2b cells, human bronchial epithelial cells (Fig. 2a). ZO-1 expression was decreased at 3 h after LPS treatment. Unfortunately, because BEAS-2b cells have several technical limitations, like transfection and growth, NCI-H292 cells were utilized from now on. When the same experiment (Fig. 2a) was performed using NCI-H292 cells, human lung mucoepidermoid carcinoma cell line, the results were the same in the NCI-H292 cells as in the normal cells (data not shown). Because we wondered whether increased ZO-1 expression could affect F-actin formation and the inflammatory microenvironment in airway cells, specific si-ZO-1 RNA was utilized. LPS dramatically increased F-actin formation in cells transfected with wild-type ZO-1 construct, but not in cells transfected with si-ZO-1 RNA (Fig. 2b). This is not surprising because LPS induced F-actin rearrangement and actin assembly are important for LPS signaling^16^. In addition, ZO-1 is known to be essential for apical surface assembly, such as the organization of F-actin^17^. More interestingly, overexpressed ZO-1 proteins could increase *pro-inflammatory cytokine* gene expression; however, siRNA-ZO-1 decreased the expression of these genes dramatically (Fig. 2c). Although overexpression of ZO-1 appears to increase the level of inflammation, ZO-1 diminished the level of *pro-inflammatory cytokine* gene expression at later stages of inflammation (24 h; data not shown). This result suggests that ZO-1 expression may increase with LPS-induced inflammation in the early-phage, but increased ZO-1 suppresses LPS-induced inflammation in the late-phage. Next, we investigated which domain of the ZO-1 protein was essential for F-actin polymerization and pro-inflammatory cytokine production during *P. aeruginosa* infection, and several deletion mutants were generated (Fig. 2d)^18^. Some scientists suggest that ZO-1 can interact with ZO-2 and F-actin to control polarization^10,18^. To determine whether a domain of ZO-1 can be critical for F-actin polymerization, several ZO-1 variants and siRNA-ZO-1 were transfected transiently before LPS treatment. While wild-type ZO-1 overexpression increased LPS-induced F-actin formation, siRNA-ZO-1 significantly decreased it. To evaluate the significance of the PDZ domain of ZO-1 protein on LPS-induced F-actin polymerization, several ZO-1 variants with domain deletions were constructed, respectively (Fig. 2e). Interestingly, M1 (with the first PDZ deleted) could have a significant effect but the removal of additional PDZs or deletion of either SH3 or GuK did not have an effect after LPS treatment. These results show that the first PDZ domain of ZO-1 has a critical effect of F-actin polymerization on LPS-induced ZO-1 function.

### The first PDZ motif peptide of ZO-1 protein suppresses LPS-induced airway inflammation

**Figure 3:**
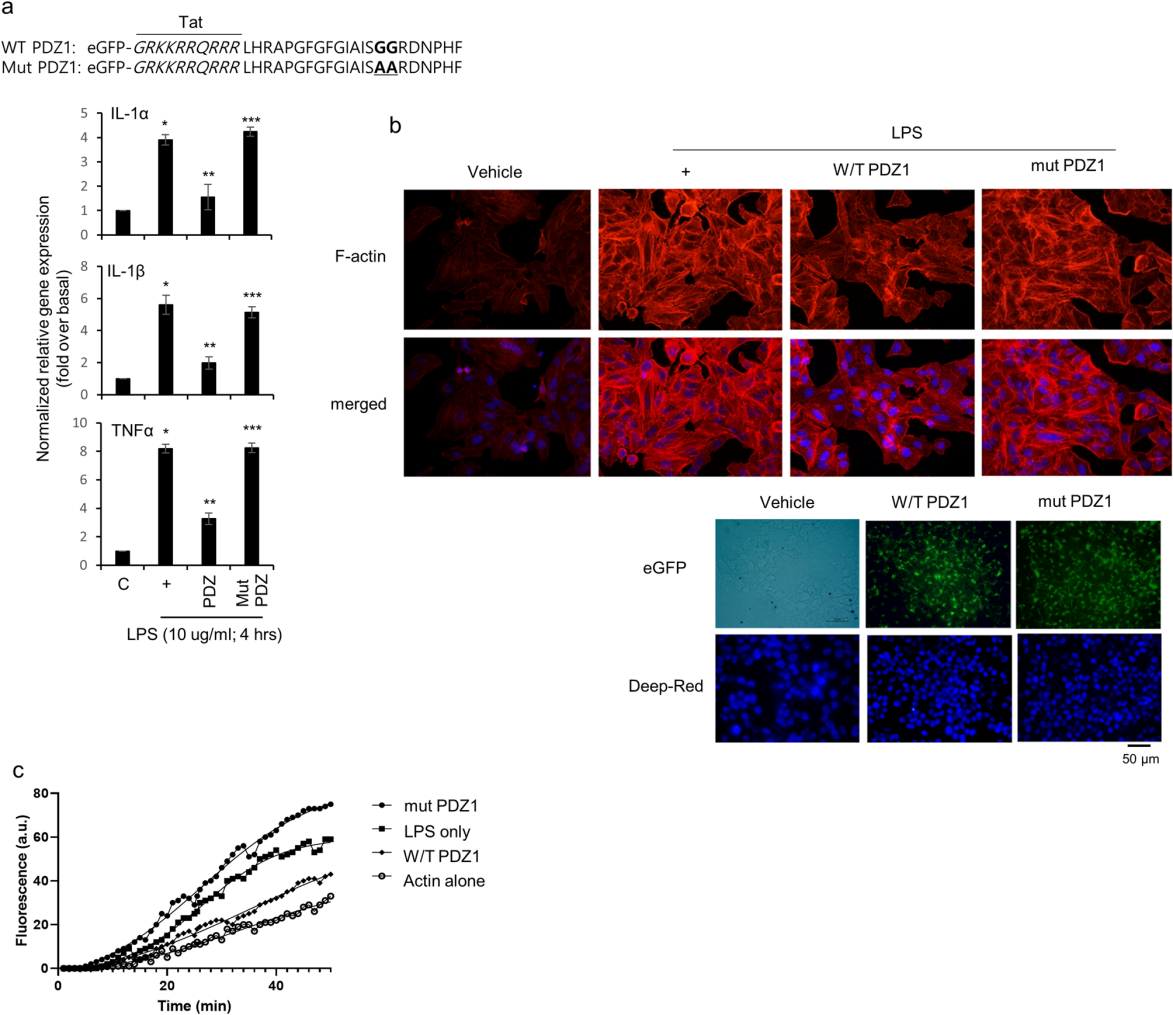
Effect of the PDZ domain of ZO-1 protein on LPS-induced airway inflammation. **(a)** Peptides were synthesized with a Tat region (italic amino acids) based on the first PDZ domain sequence (**a**, upper panel) were treated with consensus PDZ or GG25,26AA mutant PDZ peptide for 24 h and then incubated with LPS for either 4 or 10 h, after which qRT-PCR and F-actin staining (**b**, upper panel) were performed. *p < 0.05 compared with the control; **p < 0.05 compared with LPS alone; ***p < 0.05 compared with consensus PDZ peptide treatment. (**b**, lower panel), The cells were treatment with either peptide for 24 h, and then fluorescent images were captured. **(c)** The cells were treated with LPS for four hours, and cell lysates were collected, centrifuged, and then the supernatant recovered. Cell lysates with equal amounts of protein were treated with the final reaction mix containing ATP and pyrene-conjugated actin (final concentration = 0.4 mg/mL) in actin polymerization buffer. Actin polymerization was visualized by fluorescence intensity using a microplate reader with a 355 nm excitation filter and a 405 nm emission filter, and the analyses were performed using Microsoft Excel. All data shown are representative of three independent experiments.

We generated an eGFP-tagged PDZ1 peptide consisting of 22 amino acids and a TAT (GRKKRRQRRR) sequence to enable cell permeability. In addition, we generated a mutant peptide (Tat-PDZ1 GG25, 26AA) in which the important sequence of the PDZ motif (GG) was changed to AA (Fig. 3a). The efficiency of internalization of the peptides measured by eGFP were more than 80% (Fig. 3b, lower panel). Interestingly, while the consensus PDZ peptide significantly reduced F-actin polymerization, the mutant peptide increased polymerization (Fig. 3b,c). These results suggest that the PDZ peptide decreases LPS-induced F-actin polymerization, consistent with the results that the PDZ peptide down-regulates LPS-induced movement to alter inflammation at the inflamed site; however, the mutant PDZ peptide even increases F-actin polymerization or *pro-inflammatory cytokine* gene expression.

### Intracellular IL-8 secretion is induced by LPS signaling and RGS12 down-regulates the CXCR2-induced signaling pathway activated by LPS

**Figure 4:**
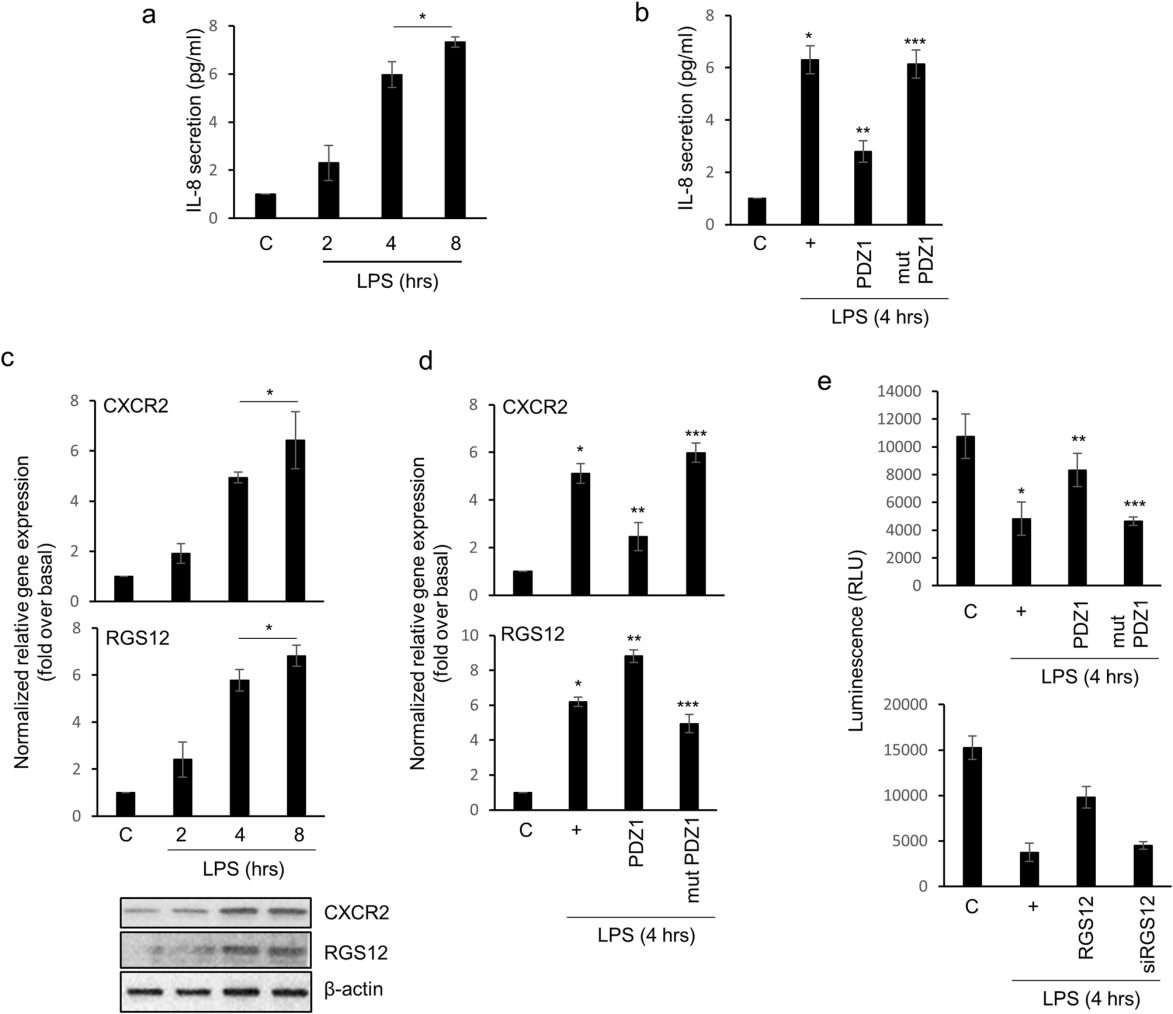
IL-8 secretion by LPS induces *CXCR2* gene expression and RGS12 inhibits CXCR2-induced signaling activated by LPS in NCI-H292 cells. **(a)** IL-8 secretion was measured by ELISA. *, *p* < 0.05 compared with control cells. **(b)** Cells were treated with consensus PDZ or mutant PDZ peptide for 24 h and then incubated with LPS for four hours. The ELISA for IL-8 was carried out with cell lysates. *p < 0.05 compared with the control; **p < 0.05 compared with LPS only; ***p < 0.05 compared with LPS and consensus PDZ peptide treatment. **(c)** Cells were incubated with LPS in a time-dependent manner, the lysates were harvested and then analyzed by qRT-PCR and Western blot. *p < 0.05 compared with the control. **(d)** Cells were treated with consensus PDZ or mutant PDZ peptide for 24 h and then incubated with LPS for four hours. Expression of CXCR2 and RGS12 was measured by qRT-PCR. *p < 0.05 compared with the control; **p < 0.05 compared with LPS only; ***p < 0.05 compared with LPS and consensus PDZ peptide treatment. (**e**, upper panel) The cells were treated with consensus PDZ or mutant PDZ peptide. After 24 h, the cells were re-trypsinized and seeded at 7000 cells/well into a 96-well plate. The cells were incubated with LPS for four hours, and a cAMP assay was performed according to the manufacturer’s instructions (cAMP-Glo assay; Promega; Madison, WI). *p < 0.05 compared with the control; **p < 0.05 compared with LPS only; ***p < 0.05 compared with LPS and consensus PDZ peptide treatment. (**e**, lower panel) The cells were transfected with an RGS12 overexpression construct or siRNA-RGS12, the cells were incubated with LPS for 4 h, and a cAMP assay was performed. *p < 0.05 compared with the control; **p < 0.05 compared with LPS only; ***p < 0.05 compared with LPS and RGS12 treatment. All data shown are representative of three independent experiments.

Next, we examined the physiological signaling mechanism by which LPS up-regulates inflammatory microenvironments in the airway. To determine whether LPS controls the secretion of cytokine/extracellular chemokine that may control the airway microenvironment, we analyzed a cytokine array with cell culture media after the treatment of cells with LPS in a time-dependent manner. Accordingly, IL-8 secretion increased in LPS-treated cell media in a dose-dependent manner (data not shown). In addition, the extracellular secretion of IL-8 obtained an extreme level after 8 h of treatment with LPS (Fig. 4a). Interestingly, the consensus PDZ peptide inhibited IL-8 secretion, but the mutant peptide did not in LPS-treated cell media (Fig. 4b). CXCR2, a G-protein-coupled receptor, and its ligand IL-8 are the most activated cytokine/chemokine in cancer and inflammation^19–21^. LPS increased *CXCR2* and the regulator of G-protein signaling 12 (*RGS12*) gene expression in a time-dependent manner (Fig. 4c). Interestingly, *CXCR2* gene expression was decreased by the consensus PDZ peptide, whereas the mutant PDZ peptide dramatically increased it (Fig. 4d). The RGS12 protein has a PDZ domain in the N-terminal region that can bind to CXCR2 ^15^. Because of the existence of the PDZ domain in RGS12, RGS12 may bind specific CXCR2 receptor and it is desensitized to shut down the signaling^11,15^. Surprisingly, the consensus PDZ peptide activated *RGS12* gene expression while reducing *CXCR2* gene expression, suggesting that the consensus PDZ peptide may control LPS-induced *CXCR2* gene expression to block its own signaling, and also increase *RGS12* gene expression as a secondary defense. To discover whether the PDZ peptide can regulate CXCR2-induced Gαi-protein signaling, the cAMP concentration was measured after LPS treatment (Fig. 4f, upper panel). The cAMP concentrations were dramatically reinstated by the consensus PDZ peptide, but not by the mutant PDZ peptide, suggesting that the consensus PDZ peptide abolished IL-8 secretion extracellularly, and inhibited its own signaling from the binding of CXCR2 and IL-8. Lastly, we examined whether the overexpression of RGS12 could also regulate LPS-induced cAMP inhibition and found that overexpressed RGS12 reinstated cAMP concentrations; however, siRNA-RGS12 did not (Fig. 4e, lower panel). These results suggest that the PDZ peptide plays a crucial negative regulator to control homeostasis during LPS-induced airway inflammation and RGS12 also functions as a secondary attenuator protein.

### The consensus PDZ peptide reduces pro-inflammatory cytokine production and decreases inflammatory cell populations in the mouse lung

**Figure 5:**
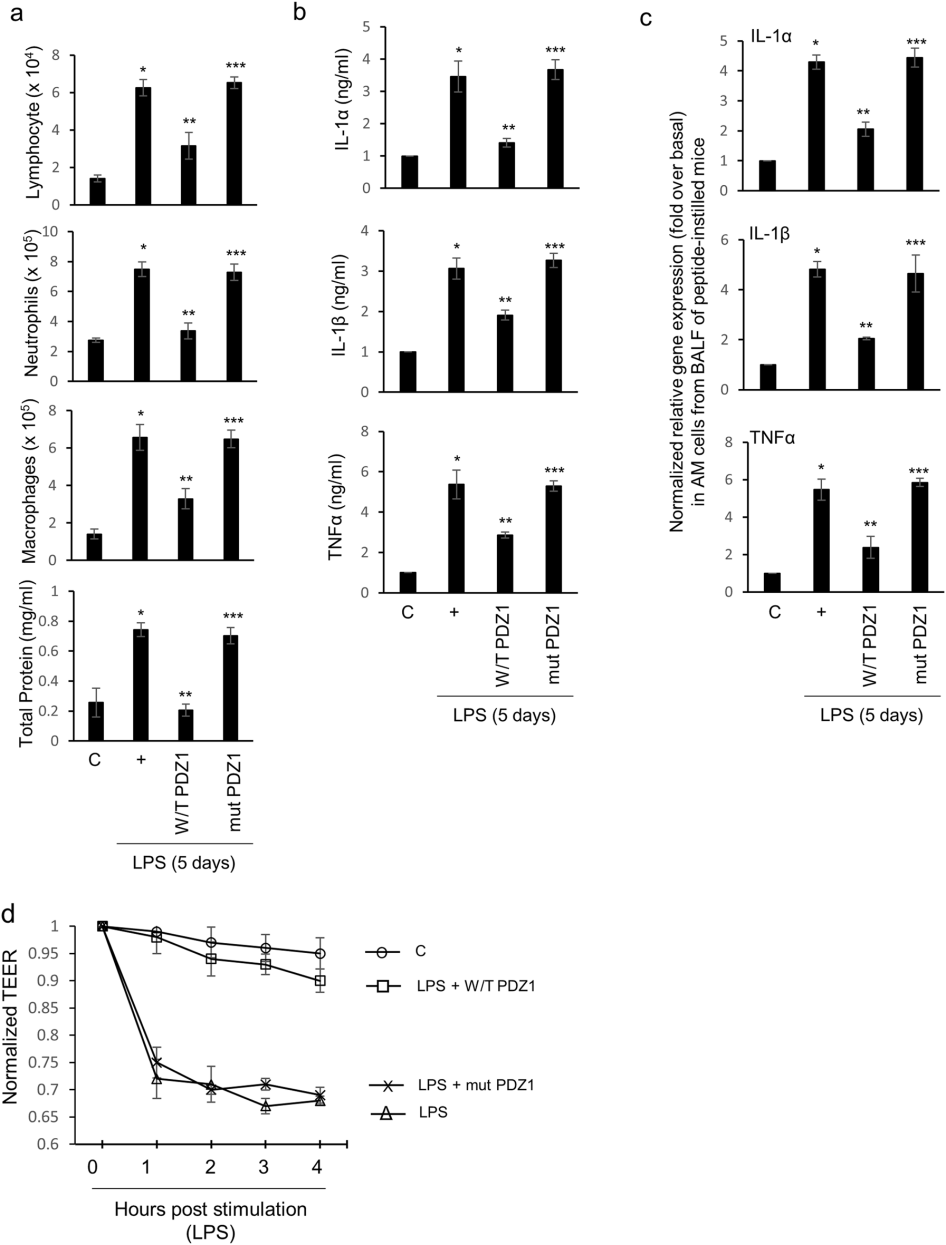
Effect of PDZ peptide on LPS-induced lung inflammatory responses in vivo. **(a)** Five days after LPS instillation (30 μl of 5 mg/kg) into the tracheal lumens of mice that had been injected with either the consensus PDZ peptide or the mutant peptide (2.0 mg/kg/30 μl) 24 h previously. The lymphocytes, neutrophils, alveolar macrophages (AMs), and total protein in BAL fluid were assessed. **(b)** The IL-6, IL-1α, IL-1β, and TNFα concentrations in the BAL fluid were measured using specific ELISAs. **(c)** AMs from the BAL fluid in healthy mice were treated with either the consensus PDZ peptide or the mutant peptide before incubation with LPS for 4 h, and then qRT-PCR was performed. *p < 0.05 compared with saline-treated mice; **p < 0.05 compared with LPS-treated mice; ***p < 0.05 compared with LPS- and consensus PDZ peptide-treated mice. **(d)** The AMs from the BAL fluid in healthy mice were treated with either the consensus PDZ peptide or the mutant peptide before incubation with LPS for various times, and then TEER testing was performed. Error bars represent the SEM of at least three independent experiments. All the data shown are representative of three independent experiments.

To examine whether the consensus PDZ peptide can control inflammatory cell populations in BAL fluid, various inflammatory cell populations were measured after intranasal instillation of the PDZ peptide before LPS exposure (Fig. 5a). The levels of lymphocytes, neutrophils, AMs, and total protein in the BAL fluid of instilled mice were decreased significantly by the consensus PDZ peptide. However, the mutant PDZ peptide increased the inflammatory cell populations dramatically in the BAL fluid of mice. Next, we investigated whether the consensus PDZ peptide also regulates the production of pro-inflammatory cytokines after LPS instillation, the levels of pro-inflammatory cytokines in BAL fluid were examined using specific ELISAs 3 days after LPS instillation (Fig. 5b). The consensus PDZ peptide also decreased LPS-induced pro-inflammatory cytokines production dramatically, whereas the mutant PDZ peptide increased the production of these cytokines. We also examined whether the PDZ peptides can control the production of pro-inflammatory cytokines in AM cells cultured from the BAL fluid of instilled mice. We found that the consensus PDZ peptide down-regulated LPS-induced airway inflammation in the lung, whereas the mutant peptide up-regulated their expression (Fig. 5c). Lastly, we found that even TEER was reinstated by the consensus PDZ peptide, but not by the mutant peptide (Fig. 5d). These results suggest that the consensus PDZ peptide eliminates changes in inflammatory cell populations, and pro-inflammatory cytokine production to maintain homeostasis in both BAL fluid and the lung after LPS treatment.

## Discussion

*P. aeruginosa* is recognized as a worldwide health hazard because it maintains infectious conditions to trigger uncontrolled inflammatory processes that lead to morbidity and death^22^. Although many new strategies to overcome *P. aeruginosa* infections and its inflammation have been attempted, *P. aeruginosa-*induced inflammation can still be robust, and antibiotic resistance even greater. Here, we provide powerful evidence that the PDZ domain of the ZO-1 tight junction protein has dual protective functions in *P. aeruginosa-*induced airway inflammation. We suggest that the PDZ peptide inhibited LPS-induced airway inflammatory phenomena and activated RGS12 protein as a secondary negative regulator against LPS signaling.

Lung inflammation induced by LPS was mediated to increase airway epithelial permeability^23^, suggesting that LPS could disrupt tight junction proteins to invade intracellularly. This result is consistent with ours (Figs. 2a, 5d, TEER). ZO-1 protein was decreased dramatically by LPS (Fig. 2a); however, claudin1 and occludin expression were increased inversely by LPS (data not shown). The exact reason why ZO-1 is decreased despite the same tight junction protein like claudin-1 or occludin is still unclear. The PDZ1 domain of ZO-1 binds directly to the C-terminal of claudins^24^, and then activates the actin polymerization at the C-termini of ZO-1, suggesting that ZO-1 may be potential regulators of claudin function in epithelial cells^25^. That the overexpression of ZO-1 could increase F-actin polymerization dramatically was understandable (Fig. 2b), because ZO-1 made cells more resistant to *P. aeruginosa* invasion*.* Overexpression of ZO-1 increased the expression of pro-inflammatory cytokine genes; while siRNA-ZO-1 decreased their expression (Fig. 2c). Unfortunately, it seems that ZO-1 may act as an inducer of inflammation. However, although overexpression of ZO-1 appears to increase the level of inflammation, ZO-1 diminished the level of pro-inflammatory cytokine gene expression at later stages of inflammation (24 h; data not shown). Thus, we thought that the reduced expression of *pro-inflammatory cytokine* genes by siRNA-ZO-1 was to prevent inflammation due to the absence of ZO-1, and it could be inferred that the inflammation was even more intense in the late-phage. These results suggest that ZO-1 may act as an attenuator in LPS-induced airway inflammation.

We also tried to identify whether the domain of ZO-1 can be essential for anti-inflammation, several ZO-1 variants and siRNA-ZO-1 were transfected transiently before LPS treatment. Our results show that the PDZ domain of the ZO-1 protein mainly decreases LPS-induced F-actin formation and airway inflammation (Fig. 2). PDZ domains are frequently found in proteins with other interaction domains or signaling domains^26^. The superfamily proteins known as membrane-associated guanylate kinases (MAGUKs) consist of one or more PDZ, SH3, and a catalytically inactive guanylate kinase-like domain; and MAGUKs include PSD-95/SAP90, Dlg, and ZO-1^27^. The multi-domain structure of PDZ-containing proteins facilitates them to network with multiple binding-partner proteins, thereby assembling larger protein complexes^28,29^. We hypothesized that (i) other binding proteins cannot be bound by the PDZ peptide, (ii) negative regulatory protein/signaling bind(s) to the PDZ peptide to stop LPS signaling, or (iii) the PDZ peptide can interrupt interactions between the PDZ-containing protein and other known/unknown proteins (or domain) to shut down LPS signaling during airway inflammation. In PDZ-containing proteins, the second PDZ domain of Na + /H + exchanger-regulating factor (NHERF) 2 binds to the C terminus of PLCβ3^30^. This interaction has PLCβ3 specific binding to NHERF2 rather than other PLC isotypes; however, the PDZ domains of ZO-1 did not bind to PLCβ3 (data not shown), suggesting that PDZ signaling induced by LPS is independent of PLCβ3 signaling in our system. Intracellular signaling pathways induced by *P. aeruginosa* infection can be estimated by identifying or characterizing other proteins (or PDZ domains) that bind to the PDZ domains of ZO-1 protein; therefore, the PDZ peptide may be a potential therapeutic candidate for *P. aeruginosa*-induced respiratory diseases.

In mammals, more than 20 members of RGS proteins are known. They contain additional motifs, such as PDZ domains, suggesting that they could mediate G proteins to other signaling pathways. In this study, we suggest that the PDZ peptide acts as a primary negative regulator to restore LPS-induced F-actin polymerization and airway inflammation. Interestingly, IL-8 was secreted extracellularly by LPS; however, IL-8 secretion was diminished by the PDZ peptide. More interestingly, CXCR2 expression activated by LPS was dramatically inhibited by PDZ, whereas, RGS12 expression increased after PDZ peptide treatment. Moreover, RGS12 restored CXCR2/IL-8-induced cAMP activity. These results show that RGS12 acts as a secondary negative regulator against LPS-induced airway inflammation. In fact, RGS12 could not only have pivotal physiological functions as a signaling regulator but was considered to be a potential therapeutic target for airborne particulate matter (PM2.5) or bacterial infection-induced airway inflammation (our unpublished data)^31^. Based on our results, we hypothesized that (i) RGS12 is activating to maintain homeostasis in inflamed conditions induced by LPS, (ii) RGS12 has a PDZ domain near the N-terminal region that recruits PDZ-binding proteins or PDZ-containing regulatory proteins such as ZO-1^32^, or (iii) RGS12 also has GPR (also known as GoLoco) motif^33^. The GPR motif binds to the Gαi protein and has GTPase activity. Therefore, RGS12 alone regulates the signaling pathway induced by LPS, and many other regulatory proteins will bind to RGS12, or various signaling mechanisms will be involved.

Based on 3D culture experiments on slides, the consensus PDZ peptide prevents LPS-induced F-actin polymerization during airway inflammation (data not shown). Accordingly, this result provides new information on the development of potential therapeutic compounds in LPS-induced respiratory diseases. We obtained evidence that the consensus PDZ peptide acted as an attenuator in vivo in the BAL fluid from LPS-instilled mice (Fig. 5). LPS from *P. aeruginosa*-induced neutrophils, lymphocytes, AMs, total protein, and pro-inflammatory cytokine expression were dramatically inhibited by the consensus PDZ peptide, whereas they were increased significantly by the mutant PDZ peptide. These results suggest that consensus PDZ peptide acts as a novel therapeutic peptide to maintain homeostasis by diminishing LPS-induced effects in vivo. The PDZ peptide also acted as a negative regulator to prevent overproduction of the pro-inflammatory cytokines in cultured AM cells in vitro. Unfortunately, it is not clear how the consensus PDZ peptide functions; however, consensus PDZ peptide may be related to apoptotic clearance in acute/chronic lung injury^34–36^.

Transepithelial electrical resistance (TEER) was used to examine the cell–cell barrier function on epithelial cells. When measuring the electrical impedance, a continuous current goes through the cells on both transcellular and paracellular pathways^37^. Specific tight junction proteins fundamentally affect epithelial resistance, thus, TEER value reflects physical structure on basolateral membrane^38,39^. Therefore, TEER measurement has been generally useful for examining the permeability of tight junctions or the membrane perturbation by toxicants on epithelial cell lines^40^. Mutant PDZ peptide dramatically abolished the permeability, whereas consensus PDZ peptide restored LPS-induced epithelial damage on epithelial cells (Fig. 5d).

We found that the consensus PDZ peptide of the ZO-1 protein could moderate LPS-induced airway inflammation and TEER. Interestingly, IL-8 secretion was intracellularly induced by treatment with LPS activated CXCR2, while RGS12 was negatively regulated with CXCR2/IL-8-induced cAMP concentrations by activating GTPase activity. The consensus PDZ peptide increased *RGS12* gene expression dramatically. In addition, PDZ induced inhibitions of the number of AMs and inflammatory cells in BAL fluid (Fig. 5). Therefore, our results suggest that the PDZ peptide may be a candidate therapeutic compound for LPS-induced pulmonary diseases in humans.

## Methods

### Materials

The LPS was purchased from Merck. Purified cytokines and specific ELISA kits were purchased from R&D Systems. The PDZ peptides were synthesized by Peptron (Daejeon, Korea). BEAS-2b and NCI-H292 cells were purchased from ATCC. Six- to eight-week-old C57BL/6 mice were maintained in accordance with the guidelines and under the approval of the Animal Care Committee of Kosin University College of Medicine, Busan, Korea.

### Lung tissue array

A human lung disease spectrum tissue array was purchased from US Biomax (Rockville, MD). The tissue array consisted of 24 cases (48 cores), which included normal lung tissue, lung hyperplasia of stroma and pulmonary fibrosis with chronic inflammation of bronchiole, lung lobar pneumonia, pulmonary atelectasis, collapsed lung, pulmonary tuberculosis, pulmonary emphysema, and inflammatory pseudotumor plus lung small cell carcinoma, lung adenocarcinomas, and lung squamous cell carcinomas. Of these, we used three different groups of tissues: normal, hyperplasia, and inflammation. Lung sections were processed with anti-ZO-1 antibody, stained with Deep-Red, and images were taken for each core. Five separate images of each section were given a score (1–7) by three independent scientists^41^.

### F-actin staining

F-actin staining was performed using ActinRed 555 ReadyProbes reagent (Molecular Probes) following the manufacturer’s instructions. Briefly, the cells were rinsed with PBS, and the ActinGreen 488 ReadyProbe reagent was added. The cells were incubated for 30 min, the stain solution removed, and the cells rinsed with PBS. Images were obtained using a Nikon Eclipse 80i microscope (Eclipse 80i) with a 488 nm excitation filter and a 532 nm emission filter.

### Actin polymerization assay

The effects of PDZ peptides on actin polymerization were examined using an Actin Polymerization Biochem Kit (Cytoskeleton; Denver, CO) following the manufacturer’s instructions. Briefly, cells were treated with LPS for 4 h, and cell lysate was collected and centrifuged at 150,000 × *g* for 1 h at 4 °C to obtain the supernatant. Cell lysates containing equal amounts of protein were treated with the final reaction mix containing ATP and pyrene-conjugated actin (final concentration = 0.4 mg/mL) in actin polymerization buffer. The kinetics of actin polymerization were visualized according to fluorescence intensity using a microplate reader with a 355 nm excitation filter and a 405 nm emission filter. All analyses were performed using Microsoft Excel.

### Transepithelial electrical resistance (TEER) testing

Before evaluation, the electrodes were sterilized and corrected according to the manufacturer’s instructions (Merck). The shorter tip was placed in the culture plate insert and the longer tip was placed in the outer well. The unit area resistance (Ω × cm^2^) was calculated by multiplying the sample resistance (Ω) by the effective area of the membrane (4.2 cm^2^ for 6-well Millicell inserts).

### Statistical analysis

Data are presented as the mean ± S.D. of at least three independent experiments. Where appropriate, statistical differences were assessed by the Wilcoxon Mann–Whitney test. *P*-values less than 0.05 were considered statistically significant (Suppl. Information).

### Ethical approval

All experimental activities were approved by the committee of Kosin University College of Medicine on Animal Resources.

## Supplementary information

Supplementary Figure S1.

## Data Availability

The datasets used and/or analyzed during the current study are available from the corresponding author on reasonable request.
