# Supplementary material for: The PDZ motif peptide of ZO-1 attenuates Pseudomonas aeruginosa LPS-induced airway inflammation
Source: Sci Rep. 2020 Nov 12;10:19644. doi: 10.1038/s41598-020-76883-9 (PMC7665049; doi:10.1038/s41598-020-76883-9)

*Supplementary Information For*

**The PDZ motif peptide of ZO-1 attenuates *Pseudomonas aeruginosa* LPS-induced airway inflammation.**

Tae Jin Lee^1^, Yung Hyun Choi^2^, and Kyoung Seob Song^3^

^1^Department of Anatomy, College of Medicine, Yeungnam University, Nam-Gu, Daegu, Korea, ^2^Department of Biochemistry, College of Korean Medicine, Dong-Eui University, Busan, Korea, ^3^Department of Cell Biology, Kosin University College of Medicine, Busan, Korea

^3^Corresponding Author

Supplementary Figure S1. (a) The full gel for Figure 2A. The gel were immunoblotted with anti-ZO-1 antibody, and then stripped and reprobed with anti-β-actin antibody. (b) The full-membrane for Fig. 2D. Anti-Flag antibody was utilized to detect overexpressed Flag-ZO-1 after transfection with several *pcDNA3.1::Flag-ZO-1* mutant constructs. And the membrane was stripped and reprobed with anti-β-actin antibody. No.6 in the upper panel was the control associated with the Western blotting anlaysis. (c) The full gel for Figure 4D. The gel were immunoblotted with anti-CXCR2 and RGS12 antibodies, and then stripped and reprobed with anti-β-actin antibody.


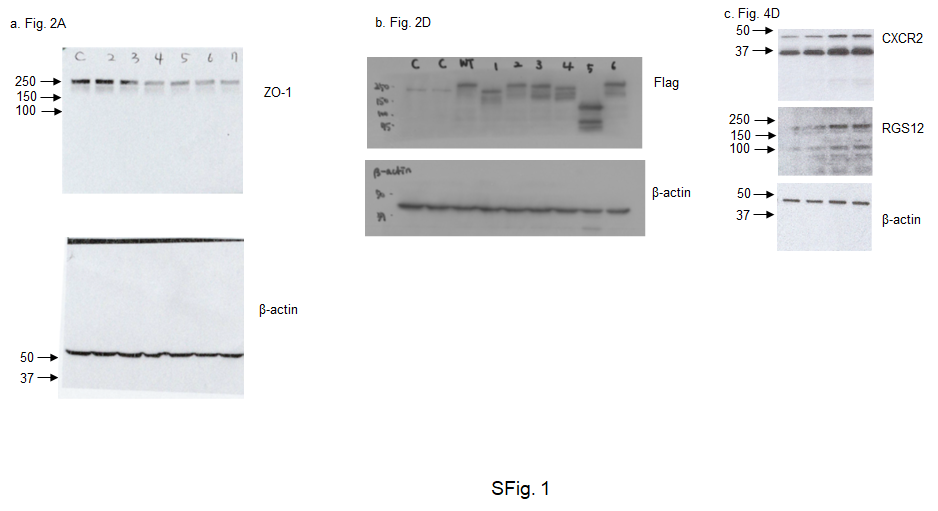

Supplement: Supplementary file 1 — Supplementary Figure S1. [file 41598_2020_76883_MOESM1_ESM.docx]
